# A LASSO-based model to predict central lymph node metastasis in preoperative patients with cN0 papillary thyroid cancer

**DOI:** 10.3389/fonc.2023.1034047

**Published:** 2023-01-25

**Authors:** Feng Zhao, Ping Wang, Chaoran Yu, Xuefei Song, Hui Wang, Jun Fang, Chenfang Zhu, Yousheng Li

**Affiliations:** ^1^ Department of General Surgery, Shanghai Ninth People’s Hospital, Shanghai Jiao Tong University School of Medicine, Shanghai, China; ^2^ Department of Ophthalmology, Shanghai Ninth People’s Hospital, Shanghai Jiao Tong University School of Medicine, Shanghai, China

**Keywords:** papillary thyroid carcinoma (PTC), central lymph node metastasis (CLNM), least absolute shrinkage and selection operator (LASSO), nomogram prediction model, clinically negative central compartment lymph nodes (cN0), prophylactic central lymph node dissection (PCLND)

## Abstract

**Introduction:**

Central lymph node metastasis (CLNM) is common in papillary thyroid carcinoma (PTC). Prophylactic central lymph node dissection (PCLND) in clinically negative central compartment lymph node (cN0) PTC patients is still controversial. How to predict CLNM before the operation is very important for surgical decision making.

**Methods:**

In this article, we retrospectively enrolled 243 cN0 PTC patients and gathered data including clinical characteristics, ultrasound (US) characteristics, pathological results of fine-needle aspiration (FNA), thyroid function, eight gene mutations, and immunoenzymatic results. Least absolute shrinkage and selection operator (LASSO) analysis was used for data dimensionality reduction and feature analysis.

**Results:**

According to the results, the important predictors of CLNM were identified. Multivariable logistic regression analysis was used to establish a new nomogram prediction model. The receiver operating characteristic (ROC) curve, calibration curve, and decision curve analysis (DCA) curve were used to evaluate the performance of the new prediction model.

**Discussion:**

The new nomogram prediction model was a reasonable and reliable model for predicting CLNM in cN0 PTC patients, but further validation is warranted.

## Introduction

Papillary thyroid carcinoma (PTC) is the most common pathological type of thyroid cancer, accounting for more than 80% of thyroid cancers (1, 2). PTC easily metastasizes to cervical lymph nodes. The rate of central lymph node metastasis (CLNM) in PTC ranges from approximately 20% to 80% (3–5). At present, the diagnosis of CLNM mainly depends on preoperative ultrasound (US), but its diagnostic sensitivity is only approximately 20–40% before surgery (6, 7). In PTC patients, positive lymph node metastasis (LNM) is associated with a worse prognosis, more advanced TNM stage, and higher rate of tumor recurrence (8, 9).

Some studies have shown that 30–40% of clinically negative central compartment lymph node (cN0) PTC patients have pathologically confirmed CLNM after prophylactic central lymph node dissection (PCLND) (10–12). However, the use of PCLND in all cN0 PTC patients is still controversial (13, 14). PCLND can improve disease-free survival and decrease the local recurrence rate (15, 16), but it also increases the rate of surgical complications, such as recurrent laryngeal nerve injury and permanent hypothyroidism (17, 18). Thus, it is necessary to predict CLNM in cN0 PTC patients to determine whether PCLND is needed.

## Materials and methods

### Patients

A total of 243 cN0 PTC patients were retrospectively enrolled in our hospital from January 2019 to July 2021. cN0 PTC patients were referred to as PTC patients without LNM under imaging examination before surgery. After surgery, all patients were divided into pathologically CLNM positive (group A) and pathologically CLNM negative (group B) groups. The clinical characteristics, ultrasound (US) characteristics, pathological fine-needle aspiration (FNA) results, thyroid function, and immunoenzymatic results were retrospectively reviewed and analysed. Paraffin-embedded PTC tissue stored in the Department of Pathology was removed for eight-gene mutation testing. The exclusion criteria were as follows: ① patients had other types of thyroid malignancy; ② patients were confirmed to have CLNM or lateral LNM before the operation; ③ patient had a history of thyroid operations; and ④ patients refused PCLND. The requirement to obtain informed consent from the patients was waived for this retrospective study.

### Operation methods

Standard operational procedures have been previously reported according to the “Chinese guidelines for diagnosis and treatment of thyroid diseases”. All patients underwent thyroidectomy with PCLND for unilateral PTC and total thyroidectomy with bilateral PCLND for bilateral PTC. All operations were performed by two experienced surgeons of the same surgery quality who had more than 10 years of working experience in our Department of General Surgery.

### Clinical and US characteristics, thyroid function, and pathological results

The clinical characteristics collected for analysis included sex (male/female), age (<55 years or ≥55 years), history of thyroid drugs (levothyroxine/thiamazole), history of Hashimoto’s thyroiditis (HT), history of nodular goiter, and family history of thyroid cancer. The US characteristics included the location of the tumor (left/right/isthmus), tumor number, tumor size, aspect ratio (≤1/>1), margin, microcalcification, capsule involvement, blood flow signal, HT, hyperthyroidism and TI-RADS classification. Thyroid function included TSH, FT3, FT4, T3, T4, TPOAb, TRAb, and TgAb. Immunohistochemistry factors included *MC, Gal-3, TTF1, TPO, Ki67*, and *CKHi*. Detailed information is provided in **Table 1**.

**Table 1:** Clinical, US, thyroid function and pathological characteristics of patients undergoing PCLND in different groups.

Variable	Group A (N=92) N (%)	Group B (N=151) N (%)	*P* value
Sex N (%)			**0.048** ^a^
Female	41 (44.6)	47 (31.1)	
Male	51 (55.4)	104 (68.9)	
Age (mean ± SD)	43.47 ± 12.37	45.45 ± 12.19	0.225
≥55	13 (14.3)	43 (28.5)	**0.017** ^a^
<55	78 (85.7)	108 (71.5)	
Thyroid-related drugs			0.714
No	91 (98.9)	147 (97.4)	
Yes	1 (1.1)	4 (2.6)	
Hashimoto’s thyroiditis			1
Positive	8 (8.7)	12 (7.9)	
Negative	84 (91.3)	139 (92.1)	
Nodular goiter			0.118
Positive	14 (15.2)	12 (7.9)	
Negative	78 (84.8)	139 (92.1)	
Family history			0.988
Positive	1 (1.1)	3 (2.0)	
Negative	91 (98.9)	148 (98.0)	
Thyroid function ^b^			
TSH			**0.024** ^a^
Positive	33 (35.9)	78 (51.7)	
Negative	59 (64.1)	73 (48.3)	
FT3			**0.016** ^a^
Positive	28 (30.4)	71 (47.0)	
Negative	64 (69.6)	80 (53.0)	
FT4			0.095
Positive	80 (87.0)	142 (94.0)	
Negative	12 (13.0)	9 (6.0)	
T3			0.417
Positive	43 (46.7)	80 (53.0)	
Negative	49 (53.3)	71 (47.0)	
T4			**0.017** ^a^
Positive	82 (89.1)	147 (97.4)	
Negative	10 (10.9)	4 (2.6)	
TgAb			0.232
Positive	27 (29.3)	57 (37.7)	
Negative	65 (70.7)	94 (62.3)	
TPOAb			0.302
Positive	23 (25.0)	63 (41.7)	
Negative	69 (75.0)	88 (58.3)	
Characteristics of US			
Bilateral			0.238
No	30 (32.6)	62 (41.1)	
Yes	62 (67.4)	89 (58.9)	
Side N (%)			0.317
Right	39 (42.4)	79 (52.3)	
Left	47 (51.1)	63 (41.7)	
Isthmus	6 (6.5)	9 (6.0)	
Tumor Size N (%)			0.074
≤10 mm	54 (58.7)	108 (72.0)	
>10 mm, ≤20 mm	29 (31.5)	35 (23.3)	
>20 mm	9 (9.8)	8 (4.7)	
Aspect Ratio N (%)			0.331
>1	68 (73.9)	121 (80.1)	
<1	24 (26.1)	30 (19.9))	
Margin			1
Circumscribed	14 (15.2)	23 (15.2)	
Indistinct	78 (84.8)	128 (84.8)	
Microcalcification			0.073
Negative	25 (27.2)	59 (39.1)	
Positive	67 (72.8)	92 (60.9)	
Capsule Involvement			0.186
Negative	73 (79.3)	130 (86.1)	
Positive	19 (20.7)	21 (13.9)	
Internal Vascularity			0.388
Negative	13 (14.1)	29 (19.3)	
Positive	79 (85.9)	121 (80.7)	
TI-RADS			**0.042** ^a^
4A/4B	18 (19.6)	49 (32.5)	
4C/5	74 (80.4)	102 (67.5)	
Immunohistochemistry			
*MC*			0.451
Negative	6 (13.0)	14 (20.3)	
Positive	40 (87.0)	55 (79.7)	
*Gal3*			0.393
Negative	8 (9.8)	8 (5.7)	
Positive	74 (90.2)	132 (94.3)	
*TTF1*			0.061
Negative	17 (23.9)	15 (12.4)	
Positive	54 (76.1)	106 (87.6)	
*TPO*			0.245
Negative	75 (96.2)	119 (90.8)	
Positive	3 (3.8)	12 (9.2)	
*Ki67* ≥0.02, <0.02			0.101
Negative	24 (33.3)	58 (46.4)	
Positive	48 (66.7)	67 (53.6)	
*CKHi*			0.965
Negative	11 (19.6)	15 (17.9)	
Positive	45 (80.4)	69 (82.1)	
Gene mutation			
*BRAF* ^V600E^			**<0.001** ^a^
Wild-type	8 (8.7)	85 (56.3)	
Mutant	84 (91.3)	66 (43.7)	
*TERT* ^C228T/250T^			0.663
Wild-type	90 (97.8)	150 (99.3)	
Mutant	2 (2.2)	1 (0.7)	
*KRAS* ^G12C/G12V/Q61R^			1
Wild-type	91 (98.9)	149 (99.3)	
Mutant	1 (1.1)	2 (0.7)	
*HRAS* ^Q61R^			1
Wild-type	92 (100)	151 (100)	
Mutant	0	0	
*NRAS* ^Q61R^			1
Wild-type	92 (100%)	151 (100%)	
Mutant	0	0	
*CCDC6*-*RET*			1
Wild-type	92 (100%)	151 (100%)	
Mutant	0	0	
*PAX8*-*PPARG*			1
Wild-type	92 (100%)	151 (100%)	
Mutant	0	0	
*EVT6*-*NTRK3*			1
Wild-type	92 (100%)	151 (100%)	
Mutant	0	0	

(a) Bold values indicate statistical significance (*P*<0.05). (b) The values of thyroid function were divided into positive and negative. Positive means outside the normal reference value range, and negative means within the normal reference value range.

### Gene mutation testing

A thyroid cancer eight-gene detection kit (Rigen Bio, China) and SLAN-96S Real-Time PCR instrument (Hong Shi, China) were used for thyroid gene mutation testing. The kit included oncogene mutations (*BRAF* ^V600E^, *HRAS* ^Q61R^, *KRAS* ^G12C/G12V/Q61R^ *NRAS* ^Q61R^, and *TERT* ^C228T/250T^) and chromosome rearrangements (*CCDC6-RET*, *PAX8-PPARG*, and *EVT6-NTRK3*). Real-time PCR technology was used to detect point mutations and fusion mutations in eight thyroid cancer-related genes.

### Statistical analysis

R (v4.1.2) and SPSS 25 were used for statistical analysis. Continuous variables with a normal distribution, such as age, tumor size and thyroid function, are presented as the mean ± standard deviation and were compared by the t test. Categorical variables, including clinical characteristics, US characteristics, pathological FNA results, thyroid function, immunoenzymatic results, and eight gene mutations, are presented as frequencies or percentages (%) and were compared by the chi-square test or Fisher’s test. We converted some continuous variables to categorical variables; for example, patient age was divided into <55 years and ≥55 years (AJCC/UICC TNM staging system version 8), tumor size was divided into ≤10 mm, 10 mm<T ≤ 20 mm, and >20 mm, and thyroid function was divided as well. All variables were analysed by least absolute shrinkage and selection operator (LASSO) analysis. The minimum error of the lambda (λ) value by the cross-validation method was calculated as the standard. The influencing factors of CLNM in cN0 PTC patients were screened out and used to establish a multifactor logistic regression model and then construct a nomogram prediction model. The RS of each patient was calculated by the mathematical formula. *P*<0.05 indicated a significant difference. Receiver operating characteristic (ROC) curves, calibration curves, and decision curve analysis (DCA) curves were used to evaluate the performance of the model.

## Result

### Clinical characteristics

There were 41 females and 51 males in group A and 47 females and 104 males in group B. The male/female ratios were 1:1.24 and 1:2.21, respectively (*P*<0.05). The average ages in groups A and B were 43.37 ± 12.37 and 45.45 ± 12.19 years, respectively (*P*<0.05). The numbers of patients <55 years old in groups A and B were 78 and 108, respectively, and those ≥55 years old were 13 and 43, respectively (*P*<0.05). A history of taking thyroid drugs, a history of HT, a history of nodular goiter, and a family history of thyroid cancer were not significantly different between groups A and B. Regarding US characteristics, only TI-RADS classification was significantly different (*P*<0.05) between groups A and B (74 cases and 102 cases, respectively). Other US features were not significantly different between groups A and B (*P*>0.05). TSH, FT3, and T4 were significantly different between groups A and B (*P*<0.05). FT4, T3, TPOAb, TRAb, and TgAb levels were not significantly different between groups A and B (*P*>0.05). Immunohistochemistry factors (*MC, Gal-3, TTF1, TPO, Ki67*, and *CKHi*) were not significantly different between groups A and B (*P*>0.05). *BRAF* ^V600E^, *KRAS* ^G12C/G12V/Q61R^, and TERT^C228T/250T^ were selected for further analysis because the number of patients with other gene mutations in groups A and B was not sufficient. The number of patients with *BRAF* ^V600E^ was significantly different between groups A and B (84 and 66 cases, 91.3% and 43.7%, *P*<0.05). *KRAS* ^G12C/G12V/Q61R^ and *TERT* ^C228T/250T^ mutations were not significantly different between the two groups (*P*>0.05) (**Table 1**).

### Predictive model construction

**Figure 1:**
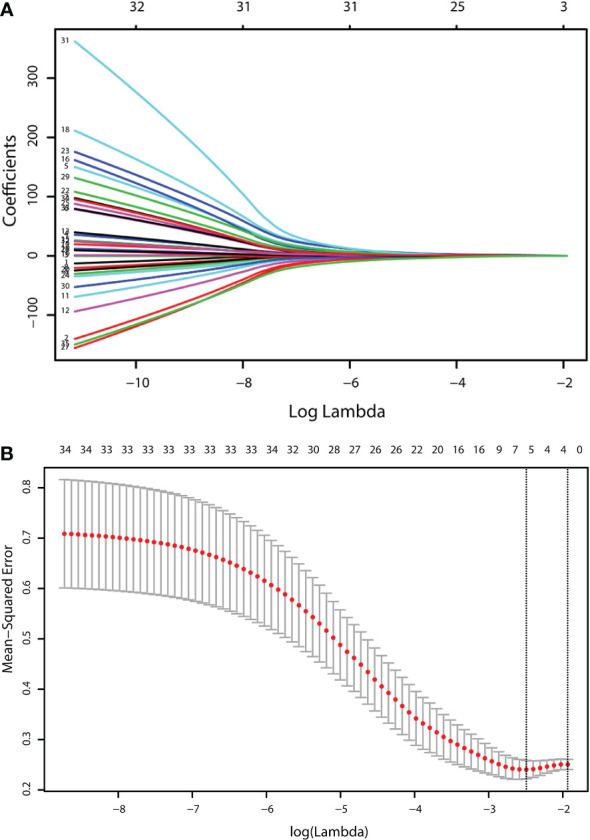
Identification of the influencing factors by LASSO regression. LASSO regression identified the following 5 most powerful predictors. **(A)** LASSO coefficient profiles of the 49 characteristics. A coefficient profile plot was produced against the log lambda (λ) sequence. **(B)** The relationship curve between the partial likelihood deviation (binomial deviation) and log(λ) was plotted.

**Figure 2:**
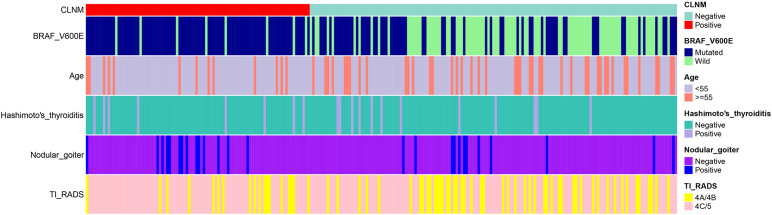
Heatmap of the top five significant variables identified based on LASSO regression analysis. Heatmap of the top five significant variables identified *via* LASSO regression, including *BRAF* ^V600E^ mutation, age, history of nodular goiter, history of Hashimoto’s thyroiditis and TI-RADS classification.

Five out of forty-nine factors, including age, history of nodular goiter, *BRAF* ^V600E^ mutation, history of HT, and TI-RADS classification, were identified as significant *via* LASSO regression (**Figure 1** and **Table 2**). Among them, *BRAF* ^V600E^ mutation was identified as the top risk factor. The statistical distribution of the five most powerful factors identified by LASSO regression analysis is visualized in **Figure 2**.

**Table 2:** Regression coefficients of the 5 most powerful factors identified by LASSO regression analysis.

Variable	OR	Std. Error	*P* value
Age (≥55)	0.317791	0.6785	0.0911
Nodular goiter	3.935329	1.2365	0.2679
*BRAF* ^V600E^	4.04959	0.6449	**0.0301^a^**
Hashimoto’s thyroiditis	1.124409	0.7038	0.8677
TI-RADS (4C/5)	1.752304	0.8284	0.4983

(a) Bold values indicate statistical significance (*P*<0.05).

**Figure 3:**
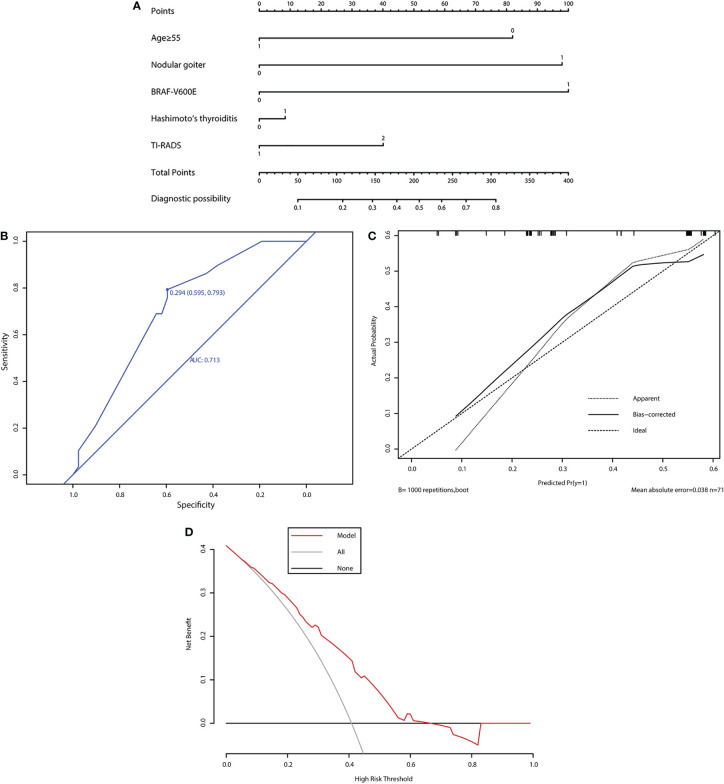
Evaluation of the performance of the new prediction model. **(A)** Nomogram for predicting CLNM in PTC patients based on five risk factors. **(B)** The ROC curve and AUC of the nomogram. ROC, receiver operating characteristic. **(C)** Calibration plots of the nomogram for predicting CLNM. **(D)** The DCA method evaluated the performance of the model.

The nomogram prediction model was constructed based on these most powerful factors (**Figure 3A**). In the nomogram prediction model, each predictive factor was assigned a corresponding score. Among the predictive factors, age ≥55 years had a score of 1, and age <55 years had a score of 0. HT, nodular goiter, and *BRAF* ^V600E^ mutation each had a score of 1 for presence and 0 for absence. The TI-RADS classification T4A~T4B was assigned a score of 1, and T4C~T5 was assigned a score of 2 (AJCC/UICC TNM staging system version 8). According to the corresponding scores from each predictive factor, the total score can be obtained. The diagnostic possibility ranges from 0.1 to 0.8. A diagnostic possibility close to 0.1 was classified as low risk, and a diagnostic possibility close to 0.8 was classified as high risk. The probability of CLNM in cN0 patients could be predicted according to the diagnostic possibility.

### Predictive model performance

The predictive model performance was evaluated by ROC curves, calibration curves, and DCA curves. The area under the ROC curve (AUC) of the model was 0.713 (95% CI 0.595–0.793) (**Figure 3B**). The approximate line and the bias-corrected line represent the performance of our model. After resampling for internal validation, the average absolute error was 3.8%. The threshold probability of CLNM metastasis was between 0.06 and 0.66. The net benefit level of the application of the nomogram prediction model was significantly higher than that of the “non-intervention” and “full intervention” schemes. Meanwhile, the calibration curve displayed a satisfactory consistency (**Figure 3C**). The DCA curve also suggested good predictive power (**Figure 3D**).

## Discussion

National Comprehensive Cancer Network (NCCN) clinical practice guidelines for thyroid carcinoma and the American Thyroid Association (ATA) guidelines do not recommend PCLND in all cN0 PTC patients (19, 20). The efficacy of PCLND for cN0 PTC is uncertain (15, 21), and the incidence of complications, such as recurrent laryngeal nerve injury, permanent hypoparathyroidism (17, 21), and chyle leakage (18), is high. In contrast, the Chinese Thyroid Association guidelines still recommend PCLND because the rate of CLNM in cN0 PTC is up to 72% (22), which increases the recurrence rate. Reoperation leads to a higher rate of operative complications (23, 24). The 2015 version of the ATA management guideline marks a high rate of CLNM as a pivotal risk factor in the risk stratification evaluation of PTC patients. Therefore, it is necessary to determine the risk of CLNM in cN0 PTC patients and screen out high-risk patients for PCLND.

US, CT, and MRI are usually used for judging the condition of central lymph nodes, but their sensitivity and specificity are not sufficient (25, 26). Zhong et al (27) showed that the incidence of CLNM in cN0 PTC patients was 53.6%. Yasuhiro et al (28) found that the sensitivity of US diagnosis of lateral metastasis was only 27.2%. For this reason, it is unreliable to perform PCLND purely depending on preoperative US. Liu et al (26) showed that there was no significant difference in the diagnosis of lateral neck node metastases between MRI and US. A meta-analysis that included 17 studies showed that the sensitivity and specificity of CT in the detection of CLNM ranged from 23% to 83% and from 64% to 94%, respectively. The pooled sensitivity was 55%, and the pooled specificity was 87% (29). Zhan et al. reported that approximately 40% of cN0 PTC patients actually had CLNM (30). In our study, the rate of CLNM in cN0 patients was 21.40%.

Some previous studies have established diagnostic models for predicting LNM in PTC. Huang et al. (31) used the LASSO method to analyze all US features and some clinical features to establish a CLNM prediction model and web-based calculator, which presented good performance. Xue et al (32) analysed the relationship between US and contrast-enhanced US characteristics and then used univariate and multivariable logistic regression methods to establish a nomogram model. Park et al (33) aimed to develop a radiomics signature using US images of the primary tumor to preoperatively predict LNM in patients with conventional PTC. Zhao et al (34) used independent predictive factors, followed by multivariate logistic regression, to evaluate risk factors by using ROC curve analysis. Most of these articles included the characteristics of US-related factors and some clinical features. Our research included all information we could obtain before surgery, such as family history of thyroid cancer, history of HT, thyroid function and gene mutations.

CLNM is a complex problem, and the use of a single variable to predict CLNM is not reliable. Multivariable regression models are commonly used to identify significant independent risk factors in medical statistical analysis. The threshold of *P*<0.05 is artificially set, and it is easy to lose some important related factors. The LASSO method does not exclude any variables that might impact the outcome, but it did not play an independent role in univariate analysis. This method was properly used to reduce the number of variables. When the weight of low correlation variables is compressed to 0, they were finally eliminated. The LASSO method is a calculation method that is more suitable for datasets that include many variables. The use of the LASSO method before logistic regression follows the “second strike theory” from the combination of generalized genetic factors and environmental factors. We believe that the combination of many factors together brought about the final clinical event of CLNM. The use of LASSO before logistic regression in the calculation process of this study took into consideration the effects of multiple factors and did not arbitrarily rule out any of the possible factors.

In our article, the rate of positive CLNM in younger patients was higher than that in elderly patients, suggesting that age was negatively correlated with CLNM, which was similar to the findings of previous studies (35, 36). Importantly, the rate of positive CLNM in patients with other adverse prognostic factors, including a history of HT, a history of nodular goiter, worse TI-RADS classification and *BRAF* ^V600E^ mutation, was higher, which indicated that these factors were positively related to CLNM. Some studies demonstrated that the *BRAF* ^V600E^ mutation was correlated with CLNM (37, 38), but others obtained the opposite conclusion (39). The relationship between the *BRAF* ^V600E^ mutation and specific clinical pathological features of PTC remains controversial. However, in our research, only the *BRAF* ^V600E^ mutation was confirmed as a significant independent risk factor for CLNM.

All the related variables were subjected to selection by the LASSO method. Using this method, we did not exclude any variables that might definitely impact the outcome, and although these factors did not have not an independent role in univariate analysis, doing this improved the pertinence and accuracy of subsequent logistic regression.

The limitations of our article are as follows. First, this was a single-centre study, which may lead to data bias. Thus, it is necessary to conduct further prospective research and multicenter studies. Finally, the evaluation of some US features is subjective, and interobserver variability may occur. Our retrospective study preliminarily explored the possibility of using certain factors to predict CLNM in cN0 PTC patients.

In conclusion, the nomogram prediction model was able to predict the risk of preoperative CLNM in cN0 PTC patients and has a good predictive performance. Further prospective, multicenter, and larger sample size studies are needed to confirm our findings.

## Data availability statement

The processed data required to reproduce these findings cannot be shared at this time as the data also forms part of an ongoing study. Requests to access the datasets should be directed to FZ, phillip_zhao@126.com.

## Ethics statement

The studies involving human participants were reviewed and approved by Ethics Committee of Shanghai Ninth People’s Hospital, Shanghai Jiao Tong University School of Medicine (SH9H-2020-T346-1). The patients/participants provided their written informed consent to participate in this study.

## Author contributions

All authors listed have made a substantial, direct, and intellectual contribution to the work, and approved it for publication.
